# Symptom-Triggered 6-Lead ECG Monitoring Significantly Increases Arrhythmia Detection Beyond Traditional Ambulatory Monitoring

**DOI:** 10.1016/j.jacadv.2026.103102

**Published:** 2026-08-19

**Authors:** Dillon J. Dzikowicz, Mehmet K. Aktas, Rebecca Horn, Bonnie MacKecknie, Kristina Cutter, Betty Mykins, Kelley Cenname, Scott McNitt, Jean-Philippe Couderc, Robert L. Strawderman, Wojciech Zareba, Jonathan S. Steinberg

**Affiliations:** a Clinical Cardiovascular Research Center, University of Rochester, Rochester, New York, USA; b School of Nursing, University of Rochester, Rochester, New York, USA; c Division of Cardiology, University of Rochester, Rochester, New York, USA; d Department of Electrical & Computer Engineering, University of Rochester, Rochester, New York, USA; e Department of Biostatistics and Computational Biology, University of Rochester, Rochester, New York, USA

**Keywords:** ambulatory, arrhythmias, atrial fibrillation, cardiac, electrocardiography, mobile applications, palpitations, patient satisfaction

## Abstract

**Background:**

Patients with palpitations and dizziness are typically monitored using ambulatory electrocardiographic (ECG) monitoring including Holter monitors and extended continuous ECG monitors. The KardiaMobile 6L (KM6L), a portable medical-grade ECG recorder, enables patients to initiate 6-lead ECG recordings during symptoms when needed.

**Objectives:**

This study aimed to assess the comparative effectiveness in detecting clinically significant arrhythmias between ambulatory ECG monitoring modalities and 30-day use of KM6L.

**Methods:**

Patients with palpitations or other arrhythmic symptoms who were referred to for standard ambulatory monitoring (either 24- or 48-hour Holter or ambulatory ECG patch) were asked to perform 1-minute, 6-lead ECG recordings with the KM6L device during symptomatic episodes over a 30-day period. Clinically significant arrhythmias were defined as atrial fibrillation/flutter, second/third-degree atrioventricular block, wide complex tachycardia, supraventricular tachycardia >100 bpm, pauses >3 seconds, or sinus tachycardia >130 bpm. Detection rates between KM6L and standard monitoring were compared using McNemar test.

**Results:**

We enrolled 350 (40%, n = 140 with 24/48-hour Holter) patients who recorded 6,251 KM6L recordings (median 15 per patient). Significant arrhythmias were detected in 66 patients (18.9%) using either ambulatory ECG monitoring or KM6L. Ambulatory ECG monitoring modalities identified arrhythmias in 27 patients (7.7%), whereas KM6L detected arrhythmias in 52 patients (14.9%) (*P* < 0.001). Importantly, 39 arrhythmia episodes (11.1%) were detected exclusively by KM6L (*P* = 0.0008), most commonly atrial fibrillation (n = 23).

**Conclusions:**

These findings demonstrate that patient-initiated, symptom-triggered ECG monitoring substantially enhances arrhythmia detection compared with traditional short-duration monitoring.

Intermittent symptoms such as palpitations, dizziness, and lightheadedness are prevalent clinical presentations that often prompt ambulatory electrocardiographic (ECG) monitoring to screen for transient cardiac arrhythmias.^1^, ^2^, ^3^ Traditional ambulatory ECG monitoring has relied on 24- or 48-hour Holter monitors, which have limited sensitivity for detecting infrequent arrhythmic events; newer patch-based ECG monitors extend ECG monitoring durations to 1 to 2 weeks resulting in higher detection sensitivity but still missing transient arrhythmia.^4^, ^5^, ^6^, ^7^, ^8^, ^9^, ^10^, ^11^

Patient-initiated intermittent ECG recordings using handheld (eg smartphone) devices could potentially improve the diagnosis of unexplained suspicious symptoms.^10^, ^11^, ^12^ These devices are widely available, can be activated quickly and easily, and are suitable for use over extended periods.^13^ Furthermore, the recorders are relatively inexpensive and require no complex electrode systems.^13^ Preliminary data suggest that smartphone-based ECG recording systems can have diagnostic yields comparable to standard event recorders.^10^^,^^11^ These devices can also be used for surveillance ECG monitoring on a fixed schedule to detect asymptomatic arrhythmic events.

The KardiaMobile 6L (KM6L) device by AliveCor is one of many commercially available ECG recording devices which uniquely offers a 6-lead ECG recording (limb leads I, II, III, aVR, aVL, and aVF) by contacting the user’s thumbs and left leg enhancing diagnostic arrhythmia recognition.^14^^,^^15^ Prior studies have not evaluated whether combining KM6L with routine Holter or patch ECG monitoring improves arrhythmia detection without overburdening the patient. In this study, we compared the detection of cardiac arrhythmias using standard-of-care 24-hour/48-hour Holter recordings or other extended ECG monitoring against arrhythmias identified through 30-day, symptom-triggered KM6L use. In addition, we sought to assess patient usability and satisfaction with the KM6L device. Accordingly, the objective of this study was not to establish diagnostic equivalence or improvement of one monitoring modality over another, but rather to compare arrhythmia detection yield under real-world clinical use, recognizing the fundamentally different acquisition paradigms of continuous passive monitoring vs intermittent, symptom-triggered ECG recording.

## Methods

### Overview

This was a prospective, single-center cohort study (Multilead Kardia ECG Evaluation of Palpitations Study) to evaluate the diagnostic utility of the KM6L device in comparison to conventional ambulatory monitoring for unexplained palpitations and related symptoms. Patients underwent both standard ambulatory ECG monitoring (Holter or extended patch ECG monitor per physician order) and concurrent 30-day monitoring with the KM6L device. The study was conducted between April 2022 and May 2023. The University of Rochester Institutional Review Board approved the study, and all patients enrolled in the study provided informed consent. No subjects withdrew from the study.

Conventional ambulatory ECG monitoring was defined as either a 24-hour or 48-hour Holter monitor (Spacelabs Healthcare) or an extended patch ECG monitor of 2-day, 7-day, or 14-day duration (BioTelemetry/Philips). The type and duration of standard monitoring was determined by the treating provider prior to enrollment, reflecting real-world practice.

### Inclusion and exclusion criteria

Patients were recruited from outpatient cardiology clinics at our institution. Adults age ≥18 who had been referred for ambulatory ECG monitoring due to unexplained palpitations, dizziness, or lightheadedness were screened for inclusion. Key inclusion criteria were ownership of a compatible smartphone (iOS or Android) to operate the Kardia app, and willingness to perform smartphone-based ECG recordings as instructed. Key exclusion criteria included: a history of unexplained syncope in the past year as these patients likely require continuous or implantable monitoring, physical or cognitive limitations preventing use of the handheld ECG (eg, severe tremor from Parkinson disease), or lack of a personal smartphone. Patients who met the criteria and agreed to participate were enrolled before or at the time of their ambulatory monitor hookup. Our target sample size was 350 patients.

### Sample size calculation

Previous studies have demonstrated the limitations of short-duration Holter monitoring in detecting clinically significant arrhythmias in symptomatic patients.^4^, ^5^, ^6^, ^7^, ^8^, ^9^ Based on these findings and to remain conservative in our estimates, we anticipate that a 48-hour Holter monitor will detect clinically relevant arrhythmias in approximately 5% to 6% of patients, whereas the KM6L device used over a 30-day period will detect arrhythmias in approximately 10% to 12% of patients, inclusive of those identified during the Holter monitoring window. Assuming a 2-sided alpha of 0.05 and aiming for 90% power to detect a difference between 5% and 10% detection rates, we estimate that a total sample size of 350 patients will be required. This calculation is consistent with sample size estimation methodology as described by Wang and Wang.^16^ Notably, given changes in practice and further use of patch-based device, we revised the protocol to include these other devices.

### Study procedure and monitoring protocols

**Figure 1:**
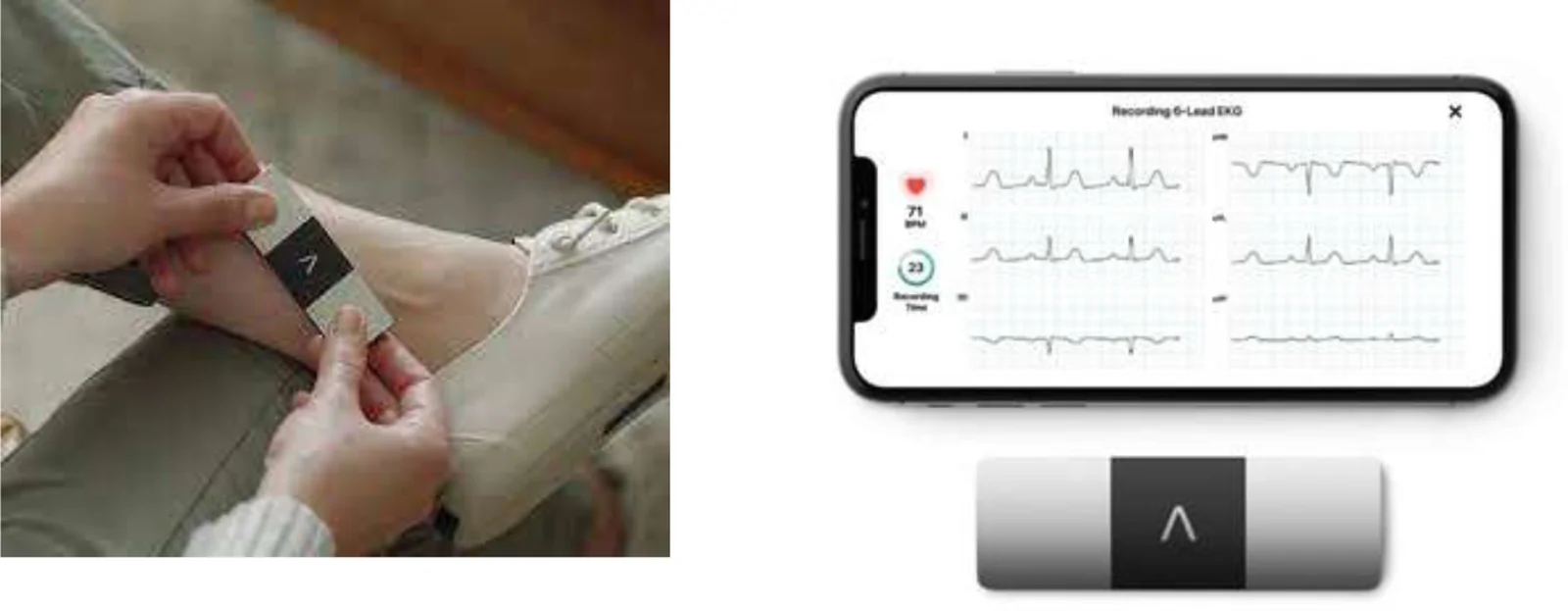
Electrocardiographic Recording Using KardiaMobile 6L and Kardia Smartphone App The KardiaMobile 6L is a portable, FDA-cleared device capable of recording a 6-lead electrocardiogram (ECG) using electrodes contacted by both thumbs and the left leg, as shown. The 6 limb leads (I, II, III, aVR, aVL, and aVF) are displayed in real-time via the smartphone app, enabling patients to capture clinically interpretable ECGs on demand during symptomatic episodes. This configuration allows for enhanced rhythm analysis and interval measurements compared to single-lead mobile ECG systems.

At the initial enrollment visit, each participant was provided with a KM6L device and given detailed instructions on its use. Research staff helped the participant install or open the Kardia app on their smartphone and demonstrated how to obtain a 6-lead, 1-minute ECG recording by placing their fingers and left leg on the device’s electrodes (Figure 1). Patients performed a supervised ECG recording during this training to ensure they could operate the device correctly. Any issues were corrected with coaching by the research staff.

Patients completed their physician-ordered ambulatory ECG monitoring as usual but were also instructed to use the KM6L device for 30 days to record ECGs whenever they experienced symptoms such as palpitations, dizziness, or other concerning cardiac sensations. To establish a baseline and ensure device familiarity, patients were additionally asked to complete scheduled asymptomatic recordings: 4 times daily (morning, midday, afternoon, and evening) during 24-hour monitoring, or 8 times daily during 48-hour or longer monitoring. They were encouraged to always carry the device with them for easy access during symptoms.

The research team performed follow-up assessments, which were conducted at 5 ± 2 days, 17 ± 2 days, and 32 ± 2 days postenrollment. At each follow-up, the research team reviewed the patients’ experience with the device and evaluated the quality of recordings. At the final follow-up visit, patients completed a questionnaire assessing ease of use and overall satisfaction with the KM6L device. Specifically, the research team asked the patients’ appreciation for the opportunity to record an ECG when needed, whether the subject would continue to use the KM6L frequently, whether the Kardia App on the subject’s own smartphone was technically easy to use, whether obtaining a 6-lead ECG recording with the left leg was easy, whether the subject was satisfied with the quality of the recordings most of the time, and whether the subject felt more confident using the KM6L to document their heart rhythm. Each question was answered across a 5-point Likert scale (1 = strongly disagree, 5 = strongly agree).

After the participant returned the ambulatory ECG monitor, the standard of care report was generated. For Holter monitors, the research technician downloaded the PDF generated report, manually extracted relevant information for this study, and cross-referenced this with the digital recording to perform additional measurements (given subsequently). For extended patch ECG monitors, a research technician downloaded the PDF generate report and manually extracted relevant information for this study.

### Quality assessment

If the participant underwent Holter monitoring, the clinical-grade digital ECG waveform data were retrieved and reviewed by the research staff. A trained research technician first examined the Holter recordings to identify any arrhythmic events, referencing the clinical report for confirmation. Subsequently, periods of temporal overlap between Holter and KM6L recordings were identified by comparing the timestamp metadata from both devices. During these overlapping intervals, the quality of each Holter ECG tracing was assessed and categorized as follows: 1) interpretable and able to make accurate heart rate and QT interval measurement; 2) interpretable but not able to make accurate heart rate and QT interval measurement; and 3) uninterpretable and not able to make accurate heart rate and QT interval measurement. All classifications were independently verified by 2 research technicians.

### Primary endpoint: arrhythmic event

The primary endpoint was the detection of clinically significant arrhythmias. Arrhythmias were defined a priori in the study protocol as atrial fibrillation (AF) or atrial flutter lasting ≥30 seconds, supraventricular tachycardia (SVT) (≥30 seconds at >130 beats/min), second- or third-degree atrioventricular block, pauses ≥3.0 seconds, or wide complex tachycardia (≥3 beats at >100 beats per minute). In addition, we included patients who had frequent premature atrial or ventricular beats. For each patient, we recorded whether a clinically significant arrhythmia was detected by the standard ambulatory monitor and/or the KM6L device. All arrhythmia classifications were independently reviewed by 2 trained research technicians and adjudicated by a board-certified cardiologist blinded to monitoring modality. The same primary endpoint definition was applied across all patients regardless of monitoring duration or modality.

### Secondary endpoints: data quality and participant satisfaction

As a secondary endpoint, we assessed the data quality of the conventional Holter and KM6L. Measurements were obtained from temporally aligned recordings on the KM6L and Holter monitors. For each participant with concurrent recordings from both devices, time-matched segments were identified using synchronized timestamps.

### Statistical analyses

Descriptive statistics were first used to characterize the overall study sample. In the primary analysis, we compared arrhythmia detection between standard-of-care ECG monitoring limited to the actual monitoring duration and 30-day use of the KM6L device. Each patient served as their own control, enabling paired comparisons of detection rates between modalities. Differences in detection were evaluated using McNemar test for paired proportions. Sensitivity analyses were conducted using the same approach, restricted to patients without a prior history of arrhythmia and separately among those undergoing extended patch ECG monitoring. Comparisons between KM6L and extended-duration patch monitoring were exploratory, not prespecified, and should be interpreted as hypothesis-generating.

Secondary analyses included 2-sided t-tests to assess demographic differences between patients with and without detected arrhythmias, as well as to compare ECG data quality between Holter monitoring and KM6L. These results are augmented with direct quantification of the degree of association between each demographic factor and the detection of arrhythmia through univariate ORs. Patient satisfaction was summarized using descriptive statistics. In addition, we evaluated the association between KM6L utilization and arrhythmia detection. The number of KM6L recordings over 30 days was compared across 3 groups: no arrhythmia, arrhythmia detected by KM6L (with or without concurrent detection by standard monitoring), and arrhythmia detected exclusively by Holter or patch monitoring. Analyses were completed in R (version 4.3.2; R Foundation for Statistical Computing) and SPSS (version 29; IBM Corp.).

## Results

A total of 350 patients (mean age 53 ± 17 years, 67% n = 234 females) were enrolled in the study. Patient demographics can be reviewed in Table 1. Overall, the sample was mid-aged, females, and White. Forty percent (n = 140) of the sample had a 24- (n = 37) or 48-hour (n = 103) Holter, whereas the remaining 60% had an extended patch ECG monitor (n = 210) which included 49.7% (n = 105) 2-day patch, 14.7% (n = 31) 7-day patch, and 35.6% (n = 74) 14-day patch. Notably, 10 patients (1 48-hour Holter, 8 2-day patch, 1 14-day patch) experienced challenges with the ambulatory monitor and a report was not generated. Nearly a quarter of patients (n = 78, 22.3%) of the sample had prior arrhythmia including AF (16%, n = 57), SVT (5%, n = 17), and wide complex tachycardia including ventricular fibrillation and ventricular tachycardia (3%, n = 11). One-quarter of the patients (n = 86, 24.6%) reported having palpitations in the past requiring cardiac monitoring. In addition to these basic comparisons, we computed univariate ORs of each demographic factor associated with detected arrhythmia regardless of modality (see Supplemental Table 1). The Central Illustration summarizes the main findings of this paper.

**Table 1:** Baseline Characteristics Stratified by Arrhythmia Detection (Any Modality)

	Total Sample (N = 350)	Without Arrhythmia (n = 284)	With Arrhythmia			*P* Value^a^
			Detected by Patch and/or KML6 (n = 27)	Detected Exclusively by KML6 (n = 39)	Overall (n = 66)	Without vs With Arrhythmia
Age, y	52.6 ± 16.5	51.1 ± 16.3	57.6 + 17.6	60.0 + 14.7	59 ± 15.9	<0.01
Body mass index, kg/m^2^	30 ± 7.6	30 ± 7.9	29.7 + 5.3	29.8 + 7.2	29.8 ± 6.5	0.87
Male	116 (33%)	90 (32%)	10 (37%)	16 (41%)	26 (39%)	0.23
White	290 (83%)	227 (80%)	24 (89%)	39 (100%)	63 (95%)	<0.01
Non-Hispanic	329 (94%)	266 (94%)	25 (93%)	38 (97%)	63 (95%)	0.33
Current/past smoking history	129 (37%)	93 (33%)	15 (56%)	21 (54%)	36 (55%)	0.001
Hypertension	144 (41%)	109 (38%)	13 (48%)	22 (56%)	35 (53%)	0.035
Diabetes mellitus	40 (12%)	28 (10%)	5 (19%)	7 (18%)	12 (18%)	0.059
Coronary artery disease	27 (8%)	21 (7%)	2 (7%)	4 (10%)	6 (9%)	0.605
Prior myocardial infarction	6 (2%)	4 (1%)	0 (0%)	2 (5%)	2 (3%)	0.318
Chronic obstructive pulmonary disease	9 (3%)	6 (2%)	1 (4%)	2 (5%)	3 (5%)	0.379
Prior stroke	5 (1%)	4 (1%)	0 (0%)	1 (3%)	1 (2%)	>0.99
AF/AFL	57 (16%)	28 (10%)	11 (41%)	19 (49%)	30 (45%)	<0.0001
SVT	17 (5%)	12 (4%)	1 (4%)	4 (10%)	5 (8%)	0.333
VT/VF	11 (3%)	11 (4%)	0 (0%)	0 (0%)	0 (0%)	0.229
Prior palpitations	86 (25%)	66 (23%)	4 (15%)	16 (41%)	20 (30%)	0.230
Prior syncope beyond 1 year	8 (2%)	6 (2%)	1 (4%)	1 (3%)	2 (3%)	0.621

Characteristics of the 350 patients who enrolled in the study, comparing those with no clinically significant arrhythmia detected (n = 284) to those in whom an arrhythmia was detected by either conventional or KardiaMobile 6L Monitoring (n = 66). Arrhythmia detection reflects clinically adjudicated events identified by any monitoring modality and does not imply equivalence of detection sensitivity.

AF/AFL = atrial fibrillation/atrial flutter; KM6L = KardiaMobile 6L; SVT = supraventricular tachycardia; VT/VF = ventricular tachycardia/ventricular fibrillation.

a *P* value represents statistical-testing between subjects with and without arrhythmia.

**Figure 2:**
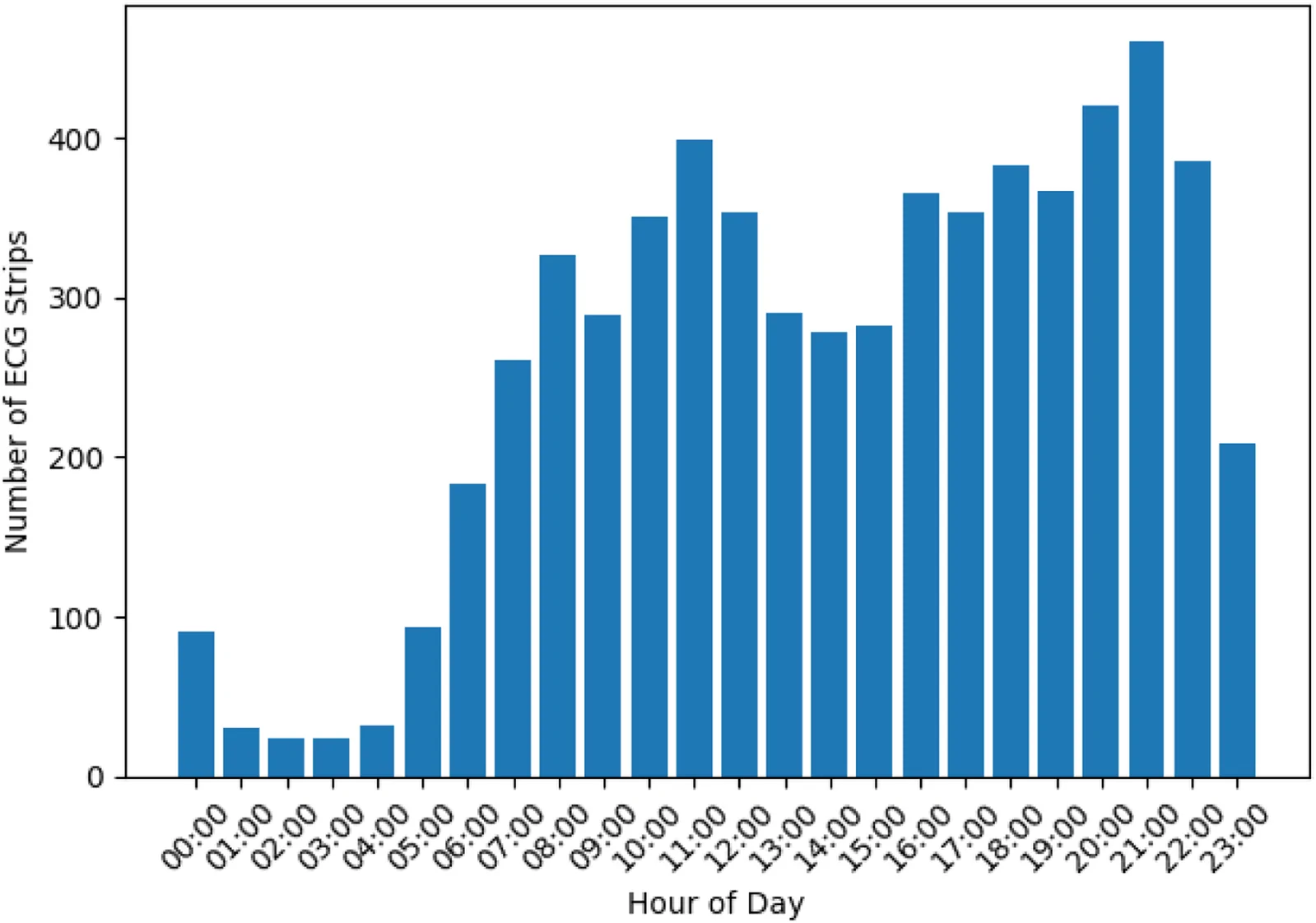
Cumulative Distribution of KM6L ECG Strips Over 24 Hours Bar plot illustrating the hourly distribution of KM6L ECG strips across a 24-hour period (00:00-23:59). The number of strips is displayed for each hour of the day, demonstrating a nonuniform temporal pattern with relatively low activity during overnight hours (00:00-05:59) and a progressive increase throughout the day, peaking in the evening hours (particularly 20:00-22:59). ECG = electrocardiogram; KM6L = KardiaMobile 6L.

A total of 6,251 KM6L recordings were collected, with a median of 15 recordings per patient (IQR: 7-150 recordings per patient). Only 7 subjects (2%) who enrolled did not complete at least 1 baseline ECG recording during the first 48 hours using KM6L, and only 3 patients failed to generate any KM6L tracings. The distribution of KM6L use can be visualized in Figure 2. There was relatively low activity during overnight hours (00:00-05:59) and a progressive increase throughout the day, peaking in the evening hours (particularly 20:00-22:59).

### Arrhythmic endpoints

**Figure 3:**
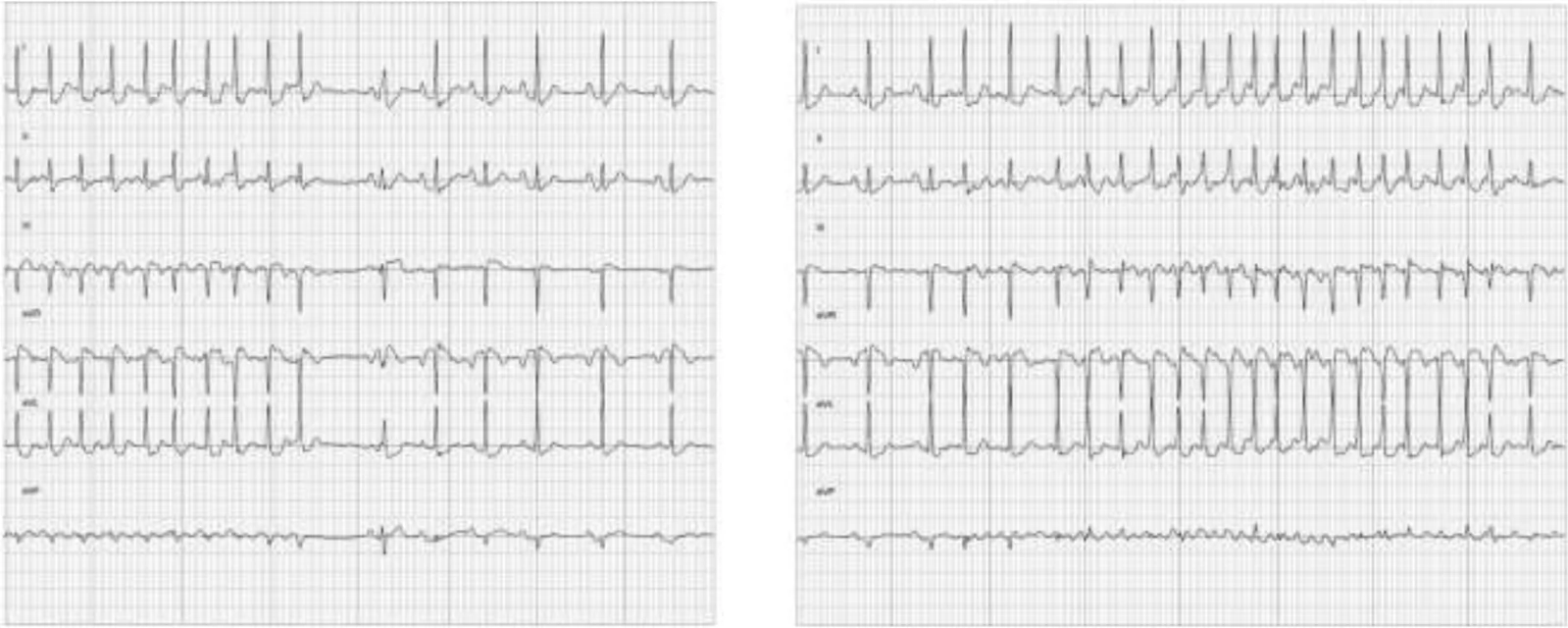
Example of Paroxysmal Atrial Fibrillation Episode Recorded by KardiaMobile 6L This 6-lead ECG tracing, acquired during a symptomatic event, demonstrates hallmark features of atrial fibrillation including an irregularly irregular rhythm and absence of distinct P waves across all leads. The tracing was recorded using the KardiaMobile 6L (AliveCor Inc), which allows patients to obtain on-demand 6-lead ECGs through thumb and left leg electrode contact. This example illustrates the device's capacity to capture clinically actionable arrhythmic events with diagnostic clarity, supporting its utility for extended ambulatory rhythm surveillance.

Clinically significant arrhythmias were detected in 66 patients (18.9%) using either KM6L or ambulatory ECG monitoring. Ambulatory ECG monitoring identified arrhythmias in 27 patients (7.7%), whereas KM6L detected arrhythmias in 52 patients (14.9%). A detailed comparison of these findings is provided in Table 2. Importantly, 39 arrhythmia episodes were detected exclusively by KM6L. KM6L detected 2 episodes of nonsustained ventricular tachycardia, 8 episodes of SVT, and 23 episodes of AF that were missed by any form of ambulatory ECG monitoring. McNemar test showed a significant difference in diagnostic yield in favor of KM6L recordings vs standard recordings (*P* = 0.0008) (Table 3). Patients with arrhythmia detected in this cohort tended to be older, more likely White, have a prior history of smoking and hypertension, and higher incidence of existing AF (*P* < 0.05) (Table 1). Taking this into consideration, we recomputed McNemar test and excluded patients with a known history of arrhythmia. In this case, ambulatory ECG monitoring identified arrhythmia in 10 patients (3.7%), and KM6L identified arrhythmia in 17 patients (6.25%) (*P* = 0. 327). Of the cases that KM6L did identify that conventional ambulatory ECG monitoring missed among patients without prior arrhythmia, KM6L detected 8 cases of AF/atrial flutter, 6 cases of SVT, 2 cases of nonsustained ventricular tachycardia, and 1 case of frequent atrial premature contractions/ ventricular premature beats. We also tested which of those patients with a history of palpitations but no known arrhythmia (n = 57), 6 had arrhythmia exclusively detected by KM6L whereas only 1 was detected by conventional ambulatory monitoring and only 2 were detected by detected by both conventional ambulatory monitoring and KM6L (*P* = 0.125). Figure 3 shows an example of paroxysmal AF recorded with KM6L device. Patients who had clinically significant arrhythmias detected by KM6L (15^14^, ^15^, ^16^, ^17^, ^18^, ^19^, ^20^, ^21^, ^22^, ^23^ tracings) did not record more tracings than those who had arrhythmia detected by ambulatory ECG monitoring only (15^11^, ^12^, ^13^, ^14^, ^15^^,^^17^ tracings) or those with no clinically significant arrhythmias (15^12^, ^13^, ^14^, ^15^^,^^17^, ^18^, ^19^, ^20^ tracings) (*P* = 0.224).

**Table 2:** KardiaMobile 6L Device Detected Significantly More Clinically Significant Cardiac Arrhythmias Than Other Ambulatory ECG Monitoring Modalities

Arrhythmia	Holter	KM6L	Both	Total
Atrial fibrillation	0	23	6	29
Atrial flutter	2	2	0	4
Supraventricular tachycardia	7	8	2	17
Nonsustained ventricular tachycardia	1	3	3	7
Frequent VPBs	2	1	3	6
Sinus tachycardia>130 bpm	0	2	0	2
Pause	1	0	0	1
Total	13	39	14	66

This table presents the number of patients in whom each type of clinically significant arrhythmia was detected, stratified by detection method: Holter only, KM6L only, or both modalities. Atrial fibrillation was the most frequently detected arrhythmia, with 29 cases, of which 23 were uniquely identified by KM6L.

ECG = electrocardiogram; VPB = ventricular premature beats; other abbreviation as in Table 1.

**Table 3:** Number of Patients With Detection of Clinically Significant Cardiac Arrhythmias by 24-48 Hour Holter Monitoring vs KardiaMobile 6 L Device

KardiaMobile 6L Result	Holter (+)	Holter (−)	Holter (NR)	Total
A) All patients, with recording status retained (N = 350)				
Positive	13	39	0	52
Negative	14	273	9	296
No recording	0	1	1	2
Total	27	313	10	350
B) All patients, with no recording classified as negative (N = 350; *P* = 0.0008)				
Positive	13	39	—	52
Negative	14	284	—	298
Total	27	323	—	350
C) Patients with no known arrhythmic history (N = 272; *P* = 0.327)				
Positive	6	16	—	16
Negative	10	239	—	256
Total	23	249	—	272

A shows a summary 3 × 3 contingency table for all 350 patients that cross-classifies patients by monitoring device, including the availability of any valid recording (NR = no recording); B shows the 2 × 2 contingency table among all patients, treating the absence of any valid recording as a nondetect (“-”), and demonstrates a significant difference in diagnostic yield in favor of KardiaMobile 6L recordings versus standard recordings (*P* value = 0.0008). C shows the 2 × 2 contingency table among patients with no known arrhythmic history and demonstrates a nonsignificant difference in diagnostic yield (*P* = 0.327).

### Arrhythmia detection by KM6L vs prolonged patch recordings

Table 4 shows comparisons of arrhythmias detected by KM6L compared to extended patch ECG monitors recordings by extended patch ECG monitors duration. There were 45 patients with arrhythmic events detected by any device. Extended patch ECG monitors devices documented arrhythmias in 17 patients (8.1%) whereas KM6L detected arrhythmias in significantly more patients: 39 (18.6%); *P* = 0.0002. When ≤7-day extended patch ECG monitors were used, McNemar test demonstrated a statistically significance difference in diagnostic yield trending toward favoring KM6L (*P* = 0.0636). With ≥7 day extended patch ECG monitors, arrhythmia was detected in 9 (9.8%) of 93 patients whereas KM6L detected arrhythmias in 22 (23.9%); *P* < 0.0001.

**Table 4:** Number of Patients With Detection of Clinically Significant Cardiac Arrhythmias by Patch ECG Recording With Different Duration vs KardiaMobile 6 L Device

KM6L Result	Patch ECG (+)	Patch ECG (−)	Total	McNemar *P* Value
All patch ECG monitors				
KM6L (+)	11	6	17	
KM6L (−)	28	165	193	
Total	39	171	210	0.0002
Extended patch ECG monitors <7 Days				
KM6L (+)	3	14	17	
KM6L (−)	5	95	100	
Total	8	109	117	0.0636
Extended patch ECG monitors ≥7 Days				
KM6L (+)	8	14	22	
KM6L (−)	1	70	71	
Total	9	84	93	<0.0001

McNemar test demonstrated a statistically significant difference in diagnostic yield favoring KM6L over prolonged EKG monitoring.

Abbreviation as in Tables 1 and 2.

### Data quality and participant satisfaction

We assessed the quality of the ECG recordings obtained from the KM6L device across the entire participant cohort. The recordings were categorized as follows: 76.5% (4,782 ECG recordings) were deemed interpretable with measurable heart rate and QT interval, 15.8% (985 ECG recordings) were interpretable but lacked measurable heart rate and QT interval and 7.7% (484 ECG recordings) were uninterpretable and lacked measurable heart rate and QT. To compare the quality of KM6L recordings with traditional Holter monitoring, we focused on patients who had time segments from both types of recordings. Among these, 88.3% (n = 757) of KM6L recordings and 86.7% of Holter recordings (n = 743) were classified as good-quality, readable ECGs.

Among the 92.3% (n = 323) of the sample that responded to our 30-day survey, 88% (n = 285) strongly agreed the KM6L app was easy to use, 70% (n = 226) strongly agreed it was easy to use KM6L with the left leg and 88% (n = 284) strongly agreed they felt confident using KM6L to document heart rhythm after 30 days. All the survey results can be Table 5.

**Table 5:** Patients Enjoyed Using KardiaMobile 6L

Questions	Median Score	Mean Score	SD	Strongly Agree (% With Score = 5)
Record ECG when need it	5.0	4.8	0.5	87%
Would you use KardiaMobile 6L frequently	5.0	4.2	1.1	51%
Technically easy to use	5.0	4.8	0.5	88%
6-lead ECG recording easy with left leg	5.0	4.5	0.9	70%
Satisfied with quality most of time	5.0	4.7	0.7	80%
Confident using KardiaMobile 6L	5.0	4.8	0.7	88%

This table summarizes responses from 323 patients who completed a post-monitoring questionnaire evaluating their experience with the KM6L device. Patients rated each statement on a 5-point Likert scale (1 = strongly disagree, 5 = strongly agree). Most patients expressed high confidence in using the device and a substantial proportion found it easy to use.

Abbreviation as in Table 2.

## Discussion

In this prospective study, we demonstrated that 30-day monitoring using the KM6L device outperformed standard short-duration ambulatory ECG monitoring alone in detecting clinically significant arrhythmias. The overall diagnostic yield of KM6L was nearly double that of standard monitoring. The incremental yield of KM6L was particularly notable among patients without a prior history of arrhythmia, whereas it identified 3 times as many clinically significant events compared to conventional monitoring. Further analysis conducted to compare diagnostic yield of 30-day use of KM6L to that of the of extended patch ECG monitors further re-enforces that clinically significant arrhythmia detection by KM6L used for 30 days compared to other, more commonly used extended patch ECG monitors. This observation further endorses the use of KM6L for detection of the causes of palpitations. These findings reinforce the value of extended, patient-initiated intermittent ECG monitoring in real-world clinical scenarios and support prior evidence that traditional Holter monitoring is limited by its brief recording window, missing most infrequent arrhythmic events.

The diagnostic yields observed in our study are consistent with the broader evidence that prolonged monitoring increases arrhythmia detection.^4^, ^5^, ^6^, ^7^, ^8^, ^9^, ^10^, ^11^ Traditional 24–48-hour Holter monitors have a relatively low yield reported at approximately 7% for patients with palpitations, largely because the monitoring window may not coincide with infrequent arrhythmic episodes.^21^ Extended monitoring can increase this marginally.^22^ In our cohort, the conventional monitors similarly captured arrhythmias in only 7.7% of patients, reflecting this limitation. By contrast, patient-activated monitoring such as the KM6L effectively extended the surveillance period beyond the constraints of a 1–2-day Holter, allowing detection of intermittent arrhythmias outside the Holter timeframe. Our findings with KM6L align with these trends but are unique in that the recordings do not require a wearable or implantable device. By only empowering patients to capture symptomatic episodes in real time, the device achieved a significantly higher diagnostic yield. This result has been reproduced by other. A prior study enrolled 625 patients with palpitations who underwent a 24-hour Holter than received a 15-day patient-activated event recording system (HeartScan) and reported significantly higher diagnostic yield with the patient-activated event record system.^23^ However, this was not a comparative study which restricts the ability to draw definitive conclusions about the relative efficacy of the patient-activated event recording system. Our study, which compared Holter and KM6L directly, provides this needed evidence. The only other comparative study we are aware of is the Catch AF trial which randomized patients 1:1 to receive either standard monitoring (resting ECG and a 24-hour Holter monitor) or enhanced monitoring (resting ECG, a 24-hour Holter monitor, and a KM6L device).^24^ In this study, arrhythmias were diagnosed in 10 patients (29%) in the standard-monitoring arm, compared to 22 patients (63%) in the enhanced-monitoring arm (*P* = 0.008).^24^ Our study corroborates these findings, showing that KM6L significantly enhances arrhythmia detection compared to standard 48-hour Holter and extended patch ECG monitors.

A key practical consideration in adopting novel monitoring tools like KM6L is the quality of data and ease of use. In our study, the majority of KM6L recordings were of diagnostic quality, indicating that the 6-lead smartphone ECG can produce clinically interpretable tracings in most cases. This high signal quality is consistent with prior validation studies of the KM6L against standard ECGs.^14^^,^^17^^,^^25^^,^^26^ For example, Azram et al^26^ demonstrated that QRS complexes recorded with the 6-lead device have 97.9% to 98.1% correlation in morphology and amplitude compared to a resting 12-lead ECG. Moreover, multiple studies have demonstrated that having 6 leads compared to a single lead improves arrhythmia detection.^14^^,^^25^ These findings suggest that the KM6L can be trusted by providers to be used by patients appropriately to capture transient arrhythmia. This is encouraging for broader implementation, because a device that is cumbersome or confusing would undermine adherence and real-world effectiveness. Moreover, the strong patient engagement seen with KM6L aligns with current clinical practice guidelines from Heart Rhythm Society and European Society of Cardiology that endorse the integration of validated consumer ECG devices into arrhythmia care.^1^^,^^27^ Our findings bolster this guideline by demonstrating that a high-quality consumer ECG device can indeed yield clinically actionable data. This aligns with the evolving landscape in electrophysiology, where wearables and handheld monitors are increasingly part of routine care decisions.^28^

Patient acceptance and usability of the KM6L were notably high. In our survey, 88% of participants *strongly agreed* that the device was easy to use, reflecting a favorable user experience even in a presumably diverse patient population. Our real-world usability data support that claims that most patients were able to successfully operate the device and obtain readable ECGs on their own. These findings are results from the iHEART trial which demonstrated that symptomology drove frequent use of patient-activated event recording systems.^29^ Although our study population included various symptomatic arrhythmias, the overarching theme is that modern ambulatory ECG technologies like KM6L can be effectively used to monitor and manage cardiac rhythm disorders outside of traditional health care settings.

Overall, the results of this study suggest that KM6L may be a suitable device to add to conventional monitoring among patients presenting with symptoms suggestive of arrhythmia (palpitations, episodic dizziness, etc). In practice, KM6L may be most effectively deployed as an adjunct early in the diagnostic pathway for patients with intermittent palpitations, potentially reducing repeated short-term monitoring and accelerating symptom-rhythm correlation. This could translate into higher detection rates of arrhythmia, and possibly faster initiation of appropriate therapies as suggested in the Catch AF trial.^24^ Although there is growth in using newer extended patch ECG monitors, it is important to note that 70% (n = 245) of all the monitors used in this study were 24- or 48-hours in duration. Thus, this is still the most common monitoring duration. Future research should compare the rate of arrhythmia between KM6L and extended patch ECG monitors (eg, 7-day, 14-day, 30-day) and time to both diagnosis and treatment using our data here as preliminary evidence. Patients presenting with palpitations may yield the highest benefit form KM6L and patient-activated event recording systems because of the intermittent nature of palpitations leading to repeated testing and delayed diagnoses.^30^ This not only prolongs patient anxiety but also increases health care expenditures.^31^^,^^32^ The KM6L offers a patient-centered solution to these challenges by enabling individuals to record ECGs during symptomatic episodes, thereby increasing the likelihood of capturing arrhythmic events without the need for repeated clinical visits.

### Study Limitations

This study has several limitations. First, it was conducted at a single center with a relatively modest sample size which may limit the power to detect infrequent arrhythmias and reduce the broader applicability of our findings. Second, the methodology relied on patient-reported symptomatic episodes to trigger KM6L recordings. Arrhythmic events that were asymptomatic or unrecognized by patients would not have been captured. Consequently, asymptomatic arrhythmias, arrhythmias that occur overnight, and situations when the user cannot use the KM6L (eg exercise) may cause underreporting. Third, the KM6L device itself has inherent constraints: it requires active patient engagement, and the quality of the ECG tracings can be variable, with occasional signal artifacts or suboptimal recordings that could affect interpretation. Finally, generalizability is limited by the need for smartphone ownership and a relatively homogeneous study population.

### Conclusions

The findings of this study indicate that the KM6L device is a tool compared to conventional 24-48-hour Holter monitoring for the detection of clinically significant arrhythmias in symptomatic patients with palpitations. The device detected a higher number of arrhythmia episodes, particularly AF, which was frequently missed by Holter monitoring. In addition, the ease of use and high patient satisfaction suggest that KM6L is well-suited for prolonged ambulatory monitoring in real-world settings. Incorporating KM6L into routine clinical practice may enhance arrhythmia detection, improve patient management, and reduce health care costs associated with undiagnosed arrhythmic events.

**Central Illustration:**
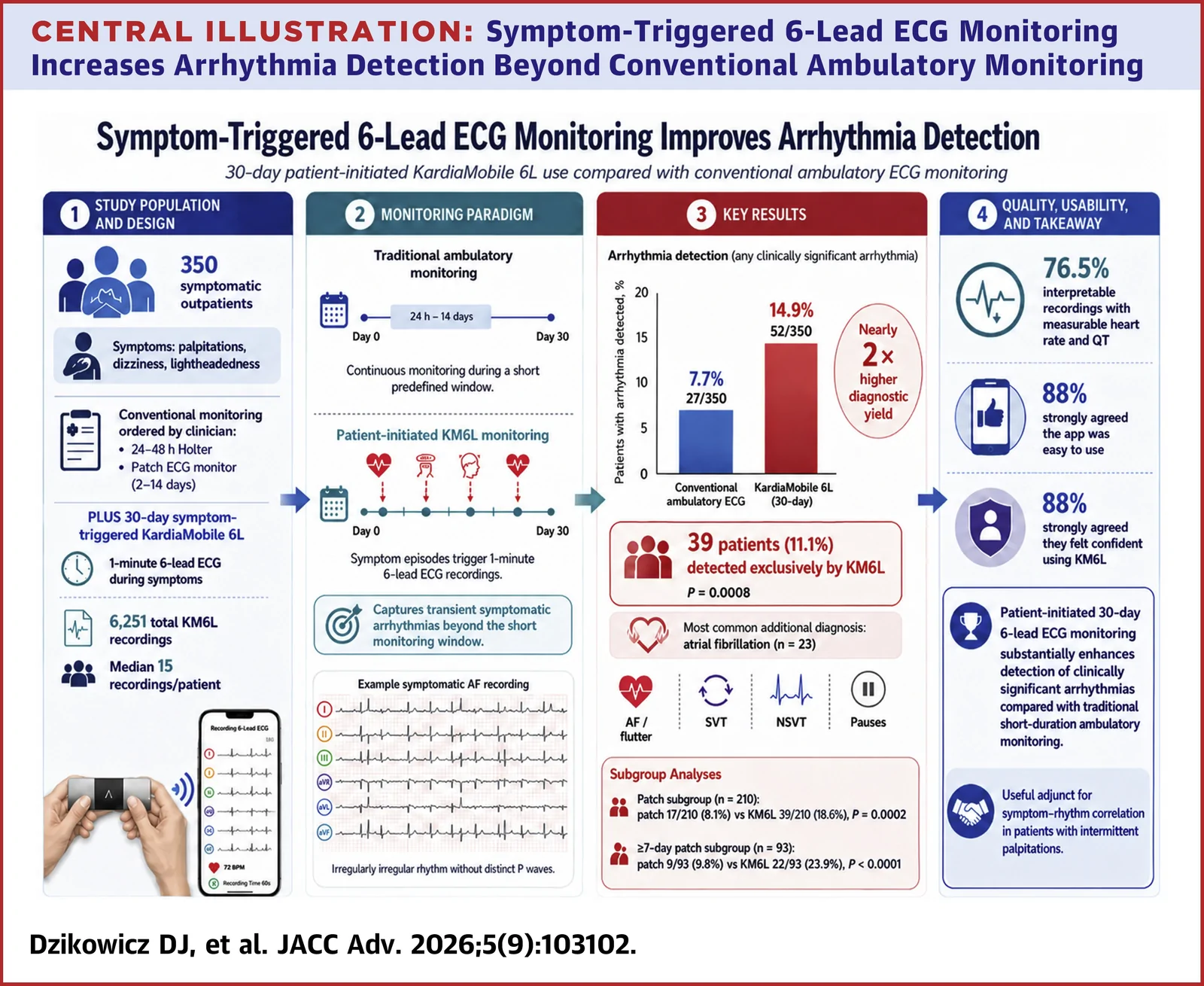
Symptom-Triggered 6-Lead ECG Monitoring Increases Arrhythmia Detection Beyond Conventional Ambulatory Monitoring In this prospective cohort of 350 adults referred for ambulatory ECG monitoring because of palpitations, dizziness, or lightheadedness, patients underwent conventional ambulatory ECG monitoring and 30-day patient-initiated KardiaMobile 6L monitoring. Conventional monitoring detected clinically significant arrhythmias in 27 patients, whereas KM6L detected arrhythmias in 52 patients. KM6L identified 39 patients with arrhythmias not detected by conventional monitoring, most commonly atrial fibrillation. The findings support symptom-triggered, handheld 6-lead ECG monitoring as a feasible adjunct for increasing arrhythmia detection in symptomatic patients. AF = atrial fibrillation; AFL = atrial flutter; ECG = electrocardiogram; KM6L = KardiaMobile 6L; NSVT = nonsustained ventricular tachycardia; SVT = supraventricular tachycardia; VPBS = ventricular premature beats; WCT= wide-complex tachycardia.

## Funding support and author disclosures

This project was funded by AliveCor (Mountain View, CA, USA). AliveCor, Inc. provided an unrestricted research grant to the University of Rochester to conduct the study. Dr Steinberg reported owning stock options in AliveCor, Inc. Dr Dzikowicz is a medical consultant for Philips North America and receives compensation from Philips North America. All other authors have reported that they have no relationships relevant to the contents of this paper to disclose.

## References

[1] Steinberg J.S., Varma N., Cygankiewicz I. (2017). 2017 ISHNE-HRS expert consensus statement on ambulatory ECG and external cardiac monitoring/telemetry. Ann Noninvasive Electrocardiol.

[2] Al-Khatib S.M., Stevenson W.G., Ackerman M.J. (2018). 2017 AHA/ACC/HRS guideline for management of patients with ventricular arrhythmias and the prevention of sudden cardiac death: a report of the American College of Cardiology/American Heart Association Task Force on Clinical Practice guidelines and the Heart Rhythm Society. Heart Rhythm.

[3] Joglar J.A., Chung M.K., Armbruster A.L. (2024). 2023 ACC/AHA/ACCP/HRS guideline for the diagnosis and management of atrial fibrillation: a report of the American College of Cardiology/American Heart Association Joint Committee on clinical practice guidelines. J Am Coll Cardiol.

[4] Kinlay S., Leitch J.W., Neil A., Chapman B.L., Hardy D.B., Fletcher P.J. (1996). Cardiac event recorders yield more diagnoses and are more cost-effective than 48-hour holter monitoring in patients with palpitations. A controlled clinical trial. Ann Intern Med.

[5] Bolourchi M., Silver E.S., Muwanga D., Mendez E., Liberman L. (2019). Comparison of Holter with patch ambulatory electrocardiographic monitoring in children: a prospective clinical trial. J Am Col Cardiol.

[6] Upadhyayula S., Kasliwal R.R. (2019). Wellysis S-PAtch cardio versus conventional holter ambulatory electrocardiographic monitoring (the PACER trial): preliminary results. J Clin Preven Cardiol.

[7] Kim J.Y., Oh I.Y., Lee H. (2023). The efficacy of detecting arrhythmia is higher with 7-day continuous electrocardiographic patch monitoring than with 24-h Holter monitoring. J Arrhythm.

[8] Chen W.C., Wu Y.L., Hsu Y.C. (2024). Comparison of continuous 24-hour and 14-day ECG monitoring for the detection of cardiac arrhythmias in patients with ischemic stroke or syncope. Clin Cardiol.

[9] Chua S.K., Chen L.C., Lien L.M. (2020). Comparison of arrhythmia detection by 24-Hour holter and 14-Day continuous electrocardiography patch monitoring. Acta Cardiol Sin.

[10] Barrett P.M., Komatireddy R., Haaser S. (2014). Comparison of 24-hour holter monitoring with 14-day novel adhesive patch electrocardiographic monitoring. Am J Med.

[11] Parikh P., Grigoriadis C.J., Dunn A., Norlock V., Wadhwa M.K. (2023). Diagnostic yield of 24-Hour holter vs 7-Day and 14-Day ePatch extended wear holter. J Am Coll Cardiol.

[12] Hendrikx T., Rosenqvist M., Wester P., Sandström H., Hörnsten R. (2014). Intermittent short ECG recording is more effective than 24-hour Holter ECG in detection of arrhythmias. BMC Cardiovasc Disord.

[13] William A.D., Kanbour M., Callahan T. (2018). Assessing the accuracy of an automated atrial fibrillation detection algorithm using smartphone technology: the iREAD study. Heart Rhythm.

[14] Bacevicius J., Taparauskaite N., Kundelis R. (2023). Six-lead electrocardiography compared to single-lead electrocardiography and photoplethysmography of a wrist-worn device for atrial fibrillation detection controlled by premature atrial or ventricular contractions: six is smarter than one. Front Cardiovasc Med.

[15] Dzikowicz D.J. (2024). A scoping review of varying Mobile electrocardiographic devices. Biol Res Nurs.

[16] Wang S.F., Wang X.R. (2013). Statistical inference of risk ratio in a correlated table with structural zero. Comput Stat.

[17] Scholten J., Jansen W.P.J., Horsthuis T. (2022). Six-lead device superior to single-lead smartwatch ECG in atrial fibrillation detection. Am Heart J.

[18] Wouters F., Gruwez H., Smeets C. (2025). Comparative evaluation of consumer wearable devices for atrial fibrillation detection: validation study. JMIR Form Res.

[19] McNemar Q. (1947). Note on the sampling error of the difference between correlated proportions or percentages. Psychometrika.

[20] Fay M.P. (2010). Two-sided exact tests and matching confidence intervals for discrete data. R J.

[21] Sulfi S., Balami D., Sekhri N. (2008). Limited clinical utility of Holter monitoring in patients with palpitations or altered consciousness: analysis of 8973 recordings in 7394 patients. Ann Noninvasive Electrocardiol.

[22] Jawad-Ul-Qamar M., Chua W., Purmah Y. (2020). Detection of unknown atrial fibrillation by prolonged ECG monitoring in an all-comer patient cohort and association with clinical and Holter variables. Open Heart.

[23] de Asmundis C., Conte G., Sieira J. (2014). Comparison of the patient-activated event recording system vs. traditional 24 h Holter electrocardiography in individuals with paroxysmal palpitations or dizziness. Europace.

[24] Coxon M.W., Hoskin K., van Zyl M., Thibert M., Sikkel M. (2024). Catch-AF-Early diagnosis of symptomatic arrythmias in the waiting period prior to seeing a cardiologist in Victoria, British Columbia. CJC Open.

[25] Metcalfe J.Z., Economou T., Naufal F. (2024). Validation of a handheld 6-Lead device for QT interval monitoring in resource-limited settings. JAMA Netw Open.

[26] Azram M., Ahmed N., Leese L. (2021). Clinical validation and evaluation of a novel six-lead handheld electrocardiogram recorder compared to the 12-lead electrocardiogram in unselected cardiology patients (EVALECG Cardio). Eur Heart J Digit Health.

[27] Masterson Creber R.M., Reading Turchioe M., Biviano A. (2022). Cardiac symptom burden and arrhythmia recurrence drives digital health use: results from the iHEART randomized controlled trial. Eur J Cardiovasc Nurs.

[28] Jensen M.T., Treskes R.W., Caiani E.G. (2021). ESC working group on e-cardiology position paper: use of commercially available wearable technology for heart rate and activity tracking in primary and secondary cardiovascular prevention-in collaboration with the European Heart Rhythm Association, European Association of Preventive Cardiology, Association of Cardiovascular Nursing and Allied Professionals, Patient Forum, and the Digital Health Committee. Eur Heart J Digit Health.

[29] Kamga P., Mostafa R., Zafar S. (2022). The use of wearable ECG devices in the clinical setting: a review. Curr Emerg Hosp Med Rep.

[30] Francisco-Pascual J., Cantalapiedra-Romero J., Pérez-Rodon J. (2021). Cardiac monitoring for patients with palpitations. World J Cardiol.

[31] Reynolds M.R., Passman R., Swindle J. (2024). Comparative effectiveness and healthcare utilization for ambulatory cardiac monitoring strategies in Medicare beneficiaries. Am Heart J.

[32] Arnold R.J., Layton A. (2015). Cost analysis and clinical outcomes of ambulatory care monitoring in Medicare patients: describing the diagnostic odyssey. J Health Econ Outcomes Res.

