# Supplementary material for: Symptom-Triggered 6-Lead ECG Monitoring Significantly Increases Arrhythmia Detection Beyond Traditional Ambulatory Monitoring
Source: JACC Adv. 2026 Aug 19;5(9):103102. doi: 10.1016/j.jacadv.2026.103102 (PMC13521091; doi:10.1016/j.jacadv.2026.103102)
Supplement: Supplementary file 1 [file mmc1.pdf]

Supplemental Table 1. Univariate Odds Ratios.

Description	B	OR	95% CI	P-Value
Age	0.032	1.033	1.014-1.051	<0.001
Male	-0.337	0.714	0.410-1.241	0.232
Body Mass Index	-0.005	0.995	0.960-1.031	0.798
White	-1.663	0.190	0.57-0.626	0.006
Non-Hispanic	-0.351	0.704	0.201-2.463	0.582
Current/Past Smoking History	0.902	2.465	1.430-4.247	0.001
Hypertension	0.595	1.813	1.057-3.108	0.031
Diabetes Mellitus	0.709	2.032	0.972-4.246	0.059
Coronary Artery Disease	0.225	1.252	0.485-3.237	0.642
Prior Myocardial Infarction	0.783	2.187	0.392-12.204	0.372
Chronic Obstructive Pulmonary Disease	0.791	2.206	0.537-9.061	0.272
Prior Stroke	-0.307	0.736	0.235-2.304	0.598
AF/AFL	2.071	7.932	4.241-14.835	<0.001
SVT	0.666	1.947	0.749-5.060	0.172
VT/VF	0.269	1.308	0.825-2.076	0.254
Prior Palpitations	0.362	1.436	0.794-2.598	0.231
Prior Syncope Beyond 1 Year	0.370	1.448	0.286-7.340	0.655
